# Ultrasound-guided non-invasive retraction for strangulated obturator hernia allows elective radical surgery: analysis of 12 cases

**DOI:** 10.1186/s40792-021-01165-z

**Published:** 2021-04-07

**Authors:** Yuto Maeda, Osamu Nakahara, Seiya Saito, Jiro Nasu, Hideo Baba

**Affiliations:** 1 Department of Gastroenterological Surgery, Graduate School of Medical Sciences, Kumamoto University, 1-1-1 Honjo, Chuo-ku, Kumamoto, 860-8556 Japan; 2 Department of Surgery, Kumamoto Rosai Hospital, 1670 Takehara-machi, Yatsushiro, 866-8533 Japan; 3 Department of Surgery, Kumamoto Chuo Hospital, 1-5-1 Tainoshima, Minami-ku, Kumamoto, 862-0965 Japan

**Keywords:** Obturator hernia, Echo-guided reduction

## Abstract

**Background:**

Obturator hernia is a life-threatening condition, requiring emergency intervention due to strangulation, if non-invasive repair for strangulation cannot be complete. Change from emergency surgery to elective surgery using minimal non-invasive options can greatly contribute to perioperative safety and curability of the underlying disease.

**Case presentation:**

12 cases of strangulated obturator hernia from April 2013 to February 2020 with male:female patient ratio of 0:12. Reduction under ultrasound guidance was possible amongst 10 out of 12 cases. The average age was 85.3 years (74–97) and average BMI was 17.4 (15.0–20.1). Based on physical findings and CT examination, diagnosis of obturator hernia was made using echo guided non-invasive reduction. Prevention in the intestinal ischemia and perforation was observed in the treated cases. Upon request, elective radical surgery was performed in 7 of these patients after their condition improved and monitored other organs for any signs.

**Conclusion:**

Attempt to improve the strangulation of obturator hernia under an echo-guided approach could enable elective and safe surgery and is believed to be a diagnostic treatment worth attempting.

## Background

Obturator hernia is a life-threatening condition, requiring emergency intervention due to strangulation, if non-invasive repair for strangulation cannot be complete. Since emergency surgery often involves laparotomy under general anesthesia, it is highly invasive for patient and may lead to a lack of manpower on the medical side. Change from emergency surgery to elective surgery using minimal non-invasive options can greatly contribute to perioperative safety and curability of the underlying disease. In this study, we evaluated the effectiveness of echo-guided reduction in strangulated obturator hernia.

## Case presentation

**Fig. 1:**
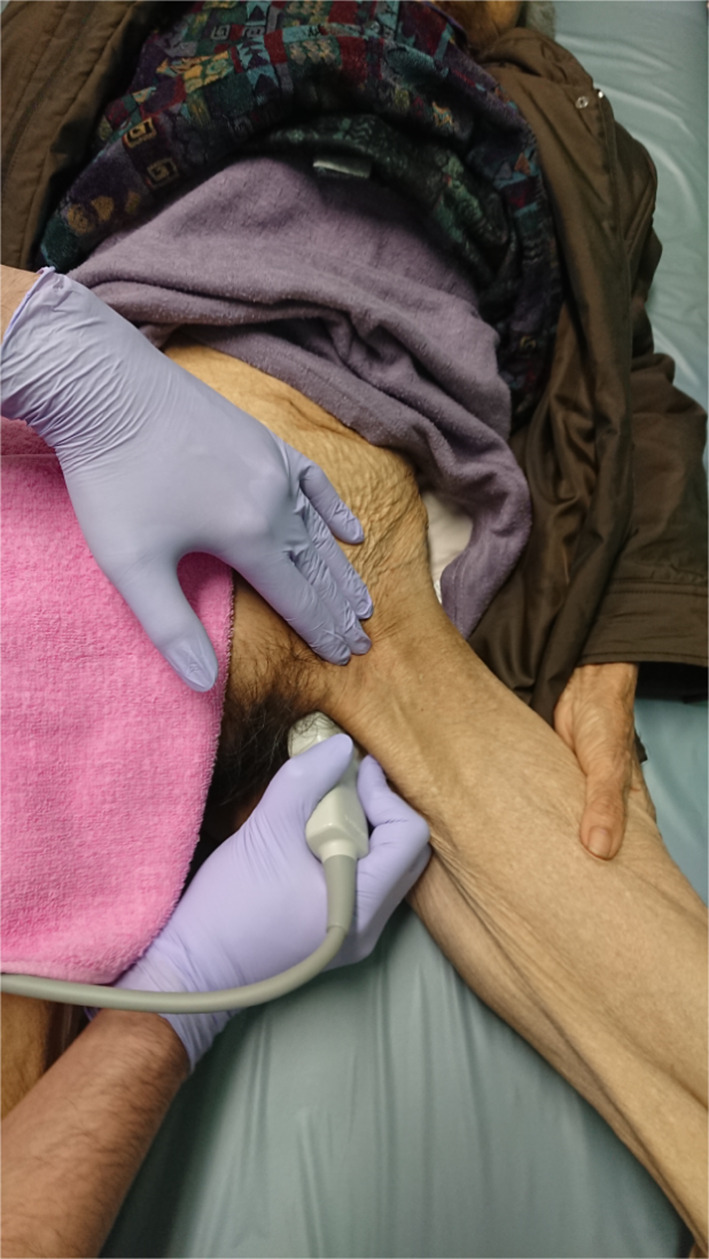
Position of repair of incarcerated obturator hernia by echo probe

**Fig. 2:**
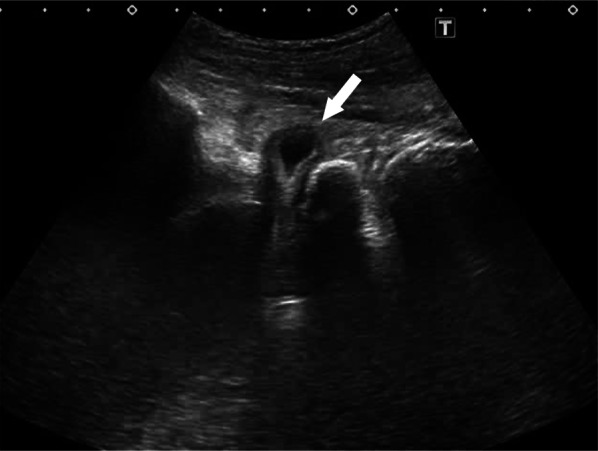
Percutaneous ultrasonographic image of incarcerated obturator hernia. The forward part of small intestine (white arrow)

**Fig. 3:**
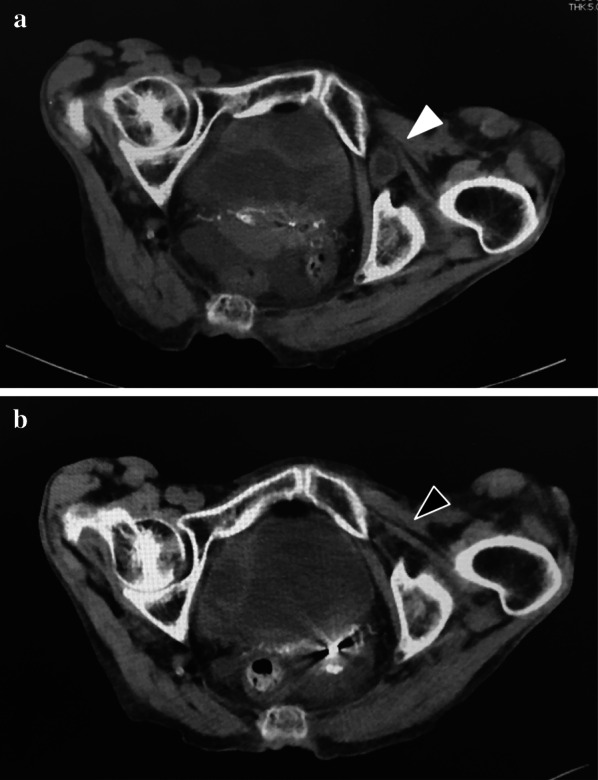
**a** CT image showed incarcerated obturator hernia. (White arrow head). **b** CT image showed disappeared incarcerated obturator hernia. (black blank arrow heads)

**Fig. 4:**
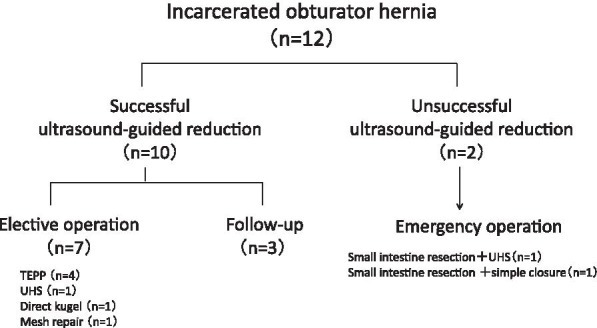
Diagram of incarcerated obturator hernia treatment

A 90-year-old woman visited a nearby physician complaining mainly of abdominal pain, constipation, vomiting, and decreased appetite. Four days later, she returned to the hospital, because her symptoms did not improve. A CT scan showed a small bowel dilatation following which she was referred to our hospital upon being diagnosed with ileus. The CT scan conducted at the referring hospital showed strangulation of the small intestine in the obturator foramen (Fig. 3a), and a non-invasive reduction was performed under echo guidance. The reduction was performed with the hip joint flexed and abducted (open/exposed) (Fig. 1). Upon examination of the groin area by echo guidance, a tumor image (strangulated intestinal tract) was confirmed near the inner side of the femoral artery (Fig. 2). The affected leg was slightly abducted and the inner side of the femoral artery was manually compressed. The index and middle fingers (or ring finger) were inserted vertically into the surface of the body. The tip of the hernia sac was identified using echo and reduced by applying pressure in the abdominal cavity while monitoring it in real-time using a probe. When the gastrointestinal tract that was incarcerated in the obturator can be reduced into the abdominal cavity under echo-guide, the disappearance of the tumor image on the echo is observed. Another CT scan was performed to confirm the reduction and the disappearance of the obturator mass (Fig. 3b). Patient follow-up was done to monitor any change in intestinal necrosis, such as peritoneal irritation, observed at the time of hospitalization. We examined 12 cases of strangulated obturator hernia from April 2013 to February 2020 with male:female patient ratio of 0:12. The average age was 85.3 years (74–97) and average BMI was 17.4 (15.0–20.1). In 9 out of 12 cases, subjective symptoms such as abdominal pain and other objective findings such as abdominal distension were apparent, and image diagnosis led to a definitive diagnosis. All patients underwent non-invasive reduction under echo guidance at the time of diagnosis of obturator hernia based on physical findings and CT examination. The average period until echo guide reduction was 3.4 days (0–8). Although there were 3 cases in which the onset mechanism was unclear, echo-guided reduction was possible in these cases as well, and no complications were observed after the reduction. Reduction under ultrasound guidance was possible amongst 10 out of 12 cases (83%). Reduction under ultrasound guidance was impossible amongst 2 out of 12 cases (17%), and required emergency surgery. The reason for the two cases of emergency surgery was not, because the perforation of the gastrointestinal tract was caused by this reduction procedure, but because there was no improvement in the findings of incarcerated obturator hernia images by reduction under the echo guide. In addition, physical findings such as abdominal distension and abdominal pain and persistent gastrointestinal dilatation findings on X-rays made it extremely dangerous to transition to strangulation ileus. Patients who could be reduced had improved abdominal symptoms without intestinal ischemia and perforation. Of the 10 cases that could be reduced, 3 cases were not surgically repaired. Two patients were 97 years and 90 years and did not undergo surgery, because the patient and family did not wish for surgical treatment. One patient was admitted to a hospital with severe cardiovascular disease. It was judged that he could not tolerate surgical invasion, and he was followed up after non-invasive retraction. Upon request, elective radical surgery was performed in 7 of these patients after their condition improved and monitored other organs for any signs. 4 patients underwent laparoscopic hernia repair under general anesthesia and three underwent anterior repair approach under lumbar spine anesthesia (Fig. 4). The average time between reduction and radical surgery was 8.5 days (2–21). Remaining 2 patients (17%) who could not undergo non-invasive reduction, underwent emergency surgery, had irreversible intestinal ischemia and required small bowel resection. Of these two patients, one underwent UHS and one underwent only a single occlusion, an obturator suture under the laparotomy with the surrounding thickened peritoneal tissue.

## Discussion

Obturator hernia is more common in thin elderly women [1]. Many cases present reduced operative resistance such as dehydration from ileus and aspiration pneumonia after vomiting. If reduction is not successful, laparotomy or intestinal resection by emergency surgery is performed even under poor general conditions. Laparotomy being extremely invasive for the elderly, increases the frequency of postoperative complications leading to prolonged hospital stay and in turn, and reduces patient's Activities of daily living(ADL) [2]. Therefore, since emergency surgery when the general condition is poor may lead to unfortunate outcomes, it is considered important to determine whether conservative treatment is possible or surgical treatment should be performed. If conservative treatment is possible, it is possible to preoperatively intervene for factors of perioperative complications (cardiovascular disease, pneumonia, and malnutrition). In strangulated obturator hernia, the elapsed time from onset of the disease till diagnosis and treatment are often unknown. In those cases, emergency surgery is necessary due to damage to the strangulated intestinal tract. However, within 12 cases of strangulated obturator hernia in our study, none of the 10 cases that could be reduced under echo guidance showed any physical findings suggesting intestinal damage and abnormal laboratory values. Conversely, the intraoperative findings of two patients whose hernia could not be reduced under echo guidance indicated that manual surgery was not possible; thus, they underwent intestinal resection. Based on the above results, the possibility of a reduction under echo guidance was the determining factor in performing emergency surgery. The onset of delayed gastrointestinal perforation after reduction was feared, so follow-up hospitalization was necessary, under the condition, where surgical intervention could be done, if needed. In all patients, we tried to perform echo-guided reduction when confirmed diagnosis of obturator hernia as the cause of abdominal pain or ileus. There is no exclusion criterion for patients who perform this echo-guided reduction, and we think that it may be performed in all cases, also as a diagnostic treatment. We think that gastrointestinal damage for allows elective surgery if reduction under echo-guide is possible, and that gastrointestinal damage for emergency surgery is occurring if reduction under echo-guide is not possible. According to successful cases of manual reduction of strangulated obturator hernia, there are cases in which manual reduction only and in which transvaginal manual reduction [3]. However, non-invasive manual reduction only may cause complications such as thigh bleeding [4]. When the diagnosis of obturator hernia was confirmed by CT, it is considered that reduction of the hernia, while confirming it in real time by echo-guide will lead to successful reduction of strangulated obturator hernia. In addition, obturator hernia is considered for emergency surgery and cannot be treated leniently. When trying with ultrasound-guided for obturator hernia, the treatment strategy is limited to 10 min per doctor, and if it could not be reduced, change to another physician and trying for an additional 10 min. If it takes longer than this, the policy is to perform emergency surgery without hesitation.

In general, laparotomy is the most common approach to radical obturator hernia surgery. However, reports of using transabdominal preperitoneal repair (TAPP) and totally extraperitoneal endoscopic repair (TEP) as the first choices are increasing [5, 6]. Compared to open surgery, laparoscopic surgery may be particularly useful for elderly people who have had relatively long periods of poor eating or ileus before surgery, in terms of insensible water loss, effects of postoperative pain on respiration and recovery of postoperative gastrointestinal motility [7]. In strangulated obturator hernia, dilation of the intestinal tract due to ileus may hinder visual field securing when performing procedures such as TAPP. Therefore, if a reduction is possible under echo guidance, normal TAPP procedures can be performed when dilatation of the intestinal tract has diminished. If reduction under echo-guide is possible, strangulated obturator hernia is effective, because the range of treatment expand; however, the possibility of emergency surgery should always be considered.

## Conclusions

Attempt to reduce the strangulation of obturator hernia under an echo-guided approach could enable elective and safe surgery and is believed to be a diagnostic treatment worth attempting.

## Data Availability

Not applicable.

## Abbreviation

CT: Computed tomography
